# From pandemic to endemic: assessing the impact of COVID-19 history and socio-demographic factors on quality of life in tuberculosis patients

**DOI:** 10.3389/fmed.2025.1582726

**Published:** 2025-06-25

**Authors:** Denisa Maria Mitroi, Mara Amalia Balteanu, Ovidiu Mircea Zlatian, Claudia Lucia Toma, Oana Maria Catana, Adina Andreea Mirea, Georgiana Camen, Viorel Biciusca, Ramona Cioboata

**Affiliations:** ^1^Doctoral School, University of Medicine and Pharmacy of Craiova, Craiova, Romania; ^2^Department of Pulmonology, Faculty of Medicine, “Titu Maiorescu” University, Bucharest, Romania; ^3^Microbiology Department, University of Medicine and Pharmacy of Craiova, Craiova, Romania; ^4^Pneumology Department, “Carol Davila” University of Medicine and Pharmacy, Bucharest, Romania; ^5^Department of Oral-Dental Prevention University of Medicine and Pharmacy of Craiova, Craiova, Romania; ^6^Radiology Department, University of Medicine and Pharmacy of Craiova, Craiova, Romania; ^7^Pneumology Department, University of Medicine and Pharmacy of Craiova, Craiova, Romania; ^8^Internal Medicine Department, “Filantropia” University Hospital, Craiova, Romania; ^9^Pneumology Department, “Victor Babes” University Hospital, Craiova, Romania

**Keywords:** tuberculosis, COVID-19, quality of life, TB relapse, socio-demographic factors

## Abstract

**Background:**

Tuberculosis (TB) and COVID-19 are major global health concerns, and their interaction, particularly regarding socio-demographic factors, remains insufficiently explored. This study assessed the impact of prior confirmed COVID-19, alongside age, education, smoking, gender, and marital status, on TB relapse risk and quality of life (QOL) among TB patients in South-Western Romania.

**Methods:**

A cross-sectional analysis was performed on 763 bacteriologically confirmed TB patients enrolled between October 2022 and January 2025. Participants provided socio-demographic and clinical information and completed the WHOQOL-BREF questionnaire. Binary logistic regression was used to identify predictors of TB relapse, while structural equation modeling (SEM) assessed factors influencing QOL.

**Results:**

Patients with a confirmed history of COVID-19 exhibited a twofold increase in TB relapse risk (OR = 2.08, *p* = 0.003). Age was a strong predictor, with individuals aged 36–60 years and those >60 years showing over fivefold (OR = 5.08, *p* < 0.001) and nearly fourfold (OR = 3.96, *p* = 0.004) increases in relapse risk, respectively. Smoking further increased relapse odds by 76% (OR = 1.77, *p* = 0.009). Conversely, secondary and tertiary education significantly reduced relapse risk (OR = 0.48, *p* = 0.002; OR = 0.46, *p* = 0.006). SEM revealed that COVID-19 history had a pronounced negative impact on QOL (β = −0.51, *p* < 0.001), while marital status (β ≈ 0.09, *p* = 0.022) and education (β ≈ 0.18, *p* < 0.001) were positively associated with QOL.

**Conclusion:**

COVID-19, advanced age, and smoking significantly elevate TB relapse risk and detrimentally affect QOL, whereas higher education appears protective. Integrated interventions addressing COVID-19 prevention, smoking cessation, and socio-economic support are essential to improve TB outcomes and patient quality of life.

## 1 Introduction

Tuberculosis (TB) and COVID-19 pandemic are two major global health concerns and have significantly impacted global health, with each disease affecting the quality of life (QOL) of patients in unique ways (1). Both are respiratory infections, and when they co-occur, they can worsen health outcomes and complicate treatment and management strategies (2).

The COVID-19 pandemic has had a profound impact on patients’ QOL, both during and after infection. Several factors, including female sex, advanced age, pre-existing comorbidities, intensive care unit (ICU) admission, and mechanical ventilation, have been associated with lower post-recovery QOL (3). Long-term sequelae following COVID-19, such as persistent fatigue, respiratory symptoms, and decreased functional status, have been widely reported and may complicate the management of chronic diseases, including tuberculosis (4). The COVID-19 pandemic officially began in late December 2019, with the first cases reported in Wuhan, China, and was declared a global pandemic by the World Health Organization (WHO) on March 11, 2020 (5). In Romania, the first confirmed case was recorded on February 26, 2020, leading to widespread public health measures, including national lockdowns and healthcare system disruptions. While the acute phase of the pandemic subsided with mass vaccination campaigns starting in late 2021, sporadic COVID-19 cases and long-term sequelae persisted through 2022 and beyond, overlapping with tuberculosis management and impacting the health status of vulnerable populations, including TB patients (6, 7).

Smoking is widely recognized as a significant risk factor for TB infection, recurrence, and poor treatment outcomes (8). Existing literature consistently demonstrates that tobacco use adversely affects immune function, increases susceptibility to *Mycobacterium tuberculosis* infection, exacerbates disease progression, and reduces treatment efficacy, substantially impairing patient QOL (9, 10). Smokers with TB exhibit higher relapse rates, more severe pulmonary impairment, and prolonged symptom resolution compared to non-smokers or former smokers. A randomized trial (11) found that quitting smoking during TB treatment significantly improved TB outcomes, including higher rates of treatment completion or cure (91% vs. 80%), lower relapse rates (6% vs. 14%), improved sputum conversion rates at week 9 (91% vs. 87%), and slightly better quality-of-life scores (EQ-5D-5L mean: 0.86 vs. 0.85). Similarly, an integrated approach combining TB directly observed therapy short-course (DOTS) with smoking cessation interventions demonstrated significantly higher improvements in health-related QOL (EQ-5D utility score: 0.98 vs. 0.91) compared to standard TB-DOTS alone (12). Moreover, the pandemic has exacerbated the challenges faced by TB patients, contributing to a significant decline in their health-related QOL (13). Increased household size, greater distance to healthcare facilities, and financial and nutritional hardships have been identified as key factors influencing this decline (14). Additionally, the disruption of healthcare services during the pandemic has resulted in reduced medication adherence among TB patients, further compromising their health-related QOL (1).

Furthermore, beyond its immediate impact, the COVID-19 pandemic disrupted efforts to prevent and control various diseases, including TB (14–16). Reports indicate that during the pandemic, the global rate of TB detection declined, while deaths related to TB increased (17).

In 2023, a total of 8.2 million people were newly diagnosed with TB worldwide, up from 7.5 million in 2022 and 7.1 million in 2019, and significantly higher than the 5.8 million and 6.4 million reported in 2020 and 2021, respectively (18). The increase in cases during 2022 and 2023 likely reflects a backlog of individuals who contracted TB in previous years but experienced delays in diagnosis and treatment due to COVID-related disruptions (19).

In 2023, TB was responsible for an estimated 1.25 million deaths (95% UI: 1.13–1.37 million), with 1.09 million occurring among HIV-negative individuals and 161,000 among people with HIV. Notably, the 1.09 million TB deaths among HIV-negative individuals were nearly twice the number of deaths attributed to HIV/AIDS (0.63 million), and TB mortality was considerably more affected by the COVID-19 pandemic than HIV/AIDS (19).

Countries with a high TB burden, such as Romania, have a substantial number of patients experiencing post-TB lung sequelae and disease relapses. According to 2023 data, Romania reported a TB incidence rate of 55 cases per 100,000 inhabitants, with an estimated 10,450 cases nationwide, marking an increase from 2020, when 8,170 cases were recorded (20).

Despite the significant burden of TB and the impact of COVID-19, there is limited research on how prior SARS-CoV-2 infection influences the QOL among TB patients, particularly in Romania (21, 22). This gap is especially evident in the South-Western region of the country, where studies on this topic remain scarce (23). Addressing this gap is essential for improving patient care and developing targeted interventions for TB patients with a history of COVID-19. Therefore, we hypothesize that the COVID-19 pandemic has had a significant impact on the health-related QOL of individuals with pulmonary TB in Romania.

## 2 Materials and methods

### 2.1 Study design and population

The objective of this study was to evaluate and compare the QOL in TB patients with and without a history of COVID-19 using a comparative cross-sectional study design.

Data collection included general demographic information and utilized the WHOQOL-BREF, a widely applied questionnaire developed by the World Health Organization (WHO) to assess quality of life across various populations and health conditions (24).

Study participants were selected from the South-Western region of Romania, with the research conducted at Victor Babeș University Hospital of Craiova. A total of 763 patients with bacteriologically confirmed pulmonary TB were enrolled in the study between October 2022 and January 2025. Participants in the study were enrolled following their voluntary informed consent, without any form of political, social, or religious discrimination, and in full accordance with the data protection legislation.

This study was conducted according to the guidelines of the Declaration of Helsinki and approved by the Institutional Review Board (or Ethics Committee) of Victor Babes University Hospital of Craiova (approval no. 24616/17.06.2022) and by the Ethics Review Board of the University Medicine and Pharmacy of Craiova (No. 408/20 November 2024).

### 2.2 Inclusion and exclusion criteria

The inclusion criteria were designed to enroll individuals aged 18 years or older who provided written informed consent and had a bacteriologically confirmed diagnosis of pulmonary TB. Participants provided information on their gender, age, education level, marital status, place of residence, disease progression, and history of retreatment.

QOL was assessed cross-sectionally upon patient enrollment into the study, conducted from October 2022 to January 2025. All participants were bacteriologically confirmed TB patients actively receiving standard anti-TB treatment as per the Romanian National TB Program guidelines, and their QOL assessments were completed within the first 2 weeks of treatment initiation, ensuring consistency in baseline data collection across the study population. Relapse was defined based on the clinical history and bacteriological confirmation at the time of the current episode, according to national TB guidelines. Participants were categorized based on their COVID-19 infection history. Patients classified as having no prior COVID-19 infection had no self-reported clinical symptoms suggestive of COVID-19, no documented positive SARS-CoV-2 PCR or antigen tests, and no clinical or epidemiological evidence of infection at the time of enrollment. Conversely, patients categorized as having a confirmed COVID-19 history had documented laboratory evidence of a positive SARS-CoV-2 PCR or antigen test, clinical confirmation of COVID-19 infection, symptom resolution, and documented completion of COVID-19-specific therapy. For these patients, the interval from recovery from COVID-19 until the QOL assessment ranged from a minimum of 3 months up to approximately 3 years, depending on the date of documented recovery from COVID-19 and study enrollment.

Patients with coexisting pulmonary diseases, including lung cancer, chronic obstructive pulmonary disease, and asthma, were excluded from the study. Individuals with mental disabilities, as well as those who were deaf or mute, were excluded from the study. The rationale for excluding deaf or mute individuals was based on practical limitations related to administering the WHOQOL-BREF questionnaire, which primarily relies on verbal interaction to accurately capture respondents’ subjective perceptions of their QOL. The potential communication barriers, even with assistance, could compromise the precision and consistency of responses, thereby affecting the validity and reliability of the collected data. Consequently, this exclusion criterion was implemented to maintain methodological uniformity and ensure the robustness of the data across all included participants.

### 2.3 WHOQOL-BREF questionnaire

The WHOQOL-BREF is a shorter version of the WHOQOL-100 and consists of 26 items, covering four key domains: physical health, psychological, social relationships, environment (24). The instrument has been used to measure the QOL of patient populations with a specific disease, including TB (25, 26).

It consists of 26 items, with the first two assessing overall QOL perception and satisfaction with health, while the remaining 24 items are categorized into four domains: physical, psychological, social, and environmental. The WHO-recommended formula was applied to calculate domain scores. Since the number of items varies across domains, each domain score was computed by multiplying the average item score by a factor of 4. The final domain scores were then normalized using the formula: (Domain Score − 4) × (100/16). Each item was rated on a 5-point Likert scale (1–5), resulting in raw domain scores ranging from 4 to 20, which were then transformed to a 0–100 scale, with higher scores indicating better QOL (24).

### 2.4 Statistical analysis

The WHOQOL-BREF instrument was used to measure health-related QOL, and domain scores were calculated following WHO guidelines. Data handling, missing value imputation, and validation procedures followed best practices to maintain the integrity and reliability of the findings.

Data was analyzed using imported into STATA 17 statistical software (StataCorp Ltd., College Station, TX, United States). Descriptive statistics were used to summarize demographic and clinical characteristics of the study participants. Continuous variables were expressed as mean ± standard deviation (SD). Categorical variables were presented as frequencies and percentages. Comparisons between groups (e.g., patients with normal and low vitamin C levels) were performed using the independent t-test for continuous variables. For categorical variables, the Fisher’s exact test was used. All statistical tests were two-tailed.

All statistical analyses were conducted using Stata 17.0 (StataCorp, College Station, TX, United States). Descriptive statistics were calculated for demographic and clinical variables, including means, standard deviations, frequencies, and proportions, where appropriate.

Binary logistic regression was performed to estimate the odds ratios (ORs) and 95% confidence intervals (CIs) for TB relapse. Independent variables included COVID-19 status, age, smoking status, gender, education, and marital status. Statistical significance was set at *p* < 0.05 for all analyses.

In addition to binary logistic regression, structural equation modeling (SEM) was employed. SEM was selected due to its ability to simultaneously estimate both direct and indirect relationships between observed and latent variables while accounting for measurement errors. The model included four latent factors representing the WHOQOL-BREF domains Physical Health, Psychological Health, Social Relationships, and Environment which were used to define a higher-order latent QOL factor. Observed predictors in the model included demographic characteristics (age, gender, marital status, and education level), clinical variables (smoking, COVID-19 history, and TB relapse), and their interactions.

The SEM was specified with standardized coefficients and estimated using maximum likelihood estimation (ML) with robust standard errors to account for potential heteroskedasticity. The model incorporated covariance structures between relevant predictor variables, ensuring a more precise estimation of latent constructs.

## 3 Results

A total of 763 TB patients were included in the analysis with 221 patients in the group without previous COVID-19 and 542 patients in the group with confirmed previous COVID-19.

### 3.1 Demographic characteristics

Among new TB cases that were smokers, the gender distribution between patients with and without previous COVID-19 infection was not significantly different (*p* = 0.129). Specifically, among patients with previous COVID-19, 29.07% were women and 70.93% were men, compared to 21.55% women and 78.45% men among those without prior COVID-19 infection. Notably, smokers with prior COVID-19 infection were significantly younger (43.36 ± 13.64 years) compared to smokers without previous COVID-19 (49.23 ± 14.19 years; *p* < 0.001). In the non-smoker group (*n* = 247), gender distribution was comparable irrespective of previous COVID-19 infection (*p* = 0.982). Specifically, women represented approximately 32.75% of cases with prior COVID-19 infection and 32.89% without. The mean ages of non-smokers with and without previous COVID-19 were similar (47.80 ± 13.92 years vs. 44.25 ± 7.87 years; *p* = 0.982), suggesting no statistically significant difference.

Regarding the patients with TB relapses, within smokers, gender distribution did not differ significantly based on previous COVID-19 infection status (*p* = 0.961), with women constituting approximately 29.11% of cases with prior COVID-19 and 28.57% without. Similarly, mean ages did not differ significantly between smokers with (52.06 ± 11.11 years) and without (49.33 ± 4.78 years) previous COVID-19 infection (*p* = 0.276). For non-smokers with TB relapse, no significant differences were observed in gender distribution (women: 35.29% with previous COVID-19 vs. 25.00% without; *p* = 0.578) or age (47.94 ± 11.37 years vs. 42.37 ± 3.99 years; *p* = 0.1832), indicating comparability across these demographic variables irrespective of COVID-19 history (Table 1).

**Table 1:** Characteristics of the patients.

New TB cases (*n* = 621)					*p* value
		Patient demographics	Previous COVID 19		
			Yes	No	
Smoking status	Smoker (*n* = 347)	Women (%)	75 (29.07%)	25 (21.55%)	0.129
		Men (%)	183 (70.93%)	91 (78.45%)	
		Age (years)	43.36 ± 13.64	49.23 ± 14.19	<0.001
	Non-smokers (*n* = 247)	Women (%)	56 (32.75%)	25 (32.89%)	0.982
		Men (%)	115 (67.25%)	51 (67.11%)	
		Age (years)	47.80 ± 13.92	44.25 ± 7.87	0.982
TB relapses (*n* = 142)					
Smoking status	Smoker (*n* = 100)	Women (%)	23 (29.11%)	6 (28.57%)	0.961
		Men (%)	56 (70.89%)	15 (71.43%)	
		Age (years)	52.06 ± 11.11	49.33 ± 4.78	0.276
	Non-smokers (*n* = 42)	Women (%)	12 (35.29%)	2 (25.00%)	0.578
		Men (%)	22 (64.71%)	6 (75.00%)	
		Age (years)	47.94 ± 11.37	42.37 ± 3.99	0.1832

*p* < 0.05: statistical significant result.

The measurements of QOL scores presented in Table 2 highlight substantial differences between TB patients stratified by previous COVID-19 infection and smoking status. Among newly diagnosed TB patients (*n* = 621), previous COVID-19 infection was significantly associated with reduced QOL across all measured domains (physical health, psychological health, social relationships, and environment) for both smokers and non-smokers (all *p*-values < 0.001). The magnitude of difference was notably higher among smokers, particularly in social relationships (−50.11%) and environmental factors (−54.45%).

**Table 2:** The quality of life scores in patients with tuberculosis, broken down by relapse and smoking status.

New TB cases (*n* = 621)						*p* value
		Quality of life domains	Previous COVID 19		Difference (%)	
			Yes	No		
Smoking status	Smoker	Physical health QOL	34.68	59.85	−42.06%	<0.001
		Psychological health QOL	36.37	54.89	−33.74%	<0.001
		Social Relationships QOL	23.55	47.2	−50.11%	<0.001
		Environment QOL	27.57	60.53	−54.45%	<0.001
	Non-smokers	Physical health QOL	47.08	63.35	−25.68%	<0.001
		Psychological health QOL	46.66	68.26	−31.64%	<0.001
		Social Relationships QOL	34.55	67.32	−48.68%	<0.001
		Environment QOL	31.07	63.53	−51.09%	<0.001
TB relapses (*n* = 142)						
Smoking status	Smoker	Physical health QOL	42.81	43.71	−2.06%	0.774
		Psychological health QOL	34.7	40.48	−14.28%	0.018
		Social Relationships QOL	41.24	50.79	−18.80%	0.017
		Environment QOL	36.83	52.38	−29.69%	<0.001
	Non-smokers	Physical health QOL	42.12	54.91	−23.29%	<0.001
		Psychological health QOL	40.2	59.37	−32.29%	<0.001
		Social Relationships QOL	29.41	57.29	−48.66%	<0.001
		Environment QOL	32.17	64.45	−50.09%	<0.001

For patients experiencing TB relapse (*n* = 142), previous COVID-19 infection demonstrated significant but varied impacts. Among smokers, the differences were statistically significant only in psychological health (−14.28%, *p* = 0.018), social relationships (−18.80%, *p* = 0.017), and notably pronounced in environmental factors (−29.69%, *p* < 0.001), but not in physical health (*p* = 0.774). Conversely, among non-smokers experiencing relapse, previous COVID-19 infection was significantly associated with substantial reductions across all QOL domains, with pronounced effects on social relationships (−48.66%) and environmental quality (−50.09%, all *p*-values < 0.001).

These findings underscore the compounding detrimental effects of previous COVID-19 infection on quality of life, particularly emphasizing greater vulnerability among smokers and the pronounced deterioration in environmental and social relational domains. Such insights could inform targeted interventions aiming to improve overall patient outcomes in post-infectious care management.

Among newly diagnosed TB patients (Table 3), previous COVID-19 infection was significantly associated with lower QOL across all age groups, particularly for younger (18–35 years) and middle-aged (36–60 years) participants, with pronounced reductions in environmental (59.09 and 53.28%, respectively; *p* < 0.001) and social relationship domains (49.63 and 50.61%; *p* < 0.001). For patients older than 60, significant declines were primarily noted in social relationships (40.74%, *p* < 0.001) and environmental domains (45.20%, *p* < 0.001).

**Table 3:** The quality of life scores in patients with tuberculosis, broken down by age group and smoking status.

New TB cases (*n* = 621)						*p* value
		Quality of life domains	Previous COVID 19		Difference (%)	
			Yes	No		
Age group	18–35 years	Physical health QOL	38.65	73.29	−47.26%	<0.001
		Psychological health QOL	40.35	66.49	−39.31%	<0.001
		Social Relationships QOL	28.47	56.52	−49.63%	<0.001
		Environment QOL	29.18	71.33	−59.09%	<0.001
	36–60 years	Physical health QOL	40.79	62.96	−35.21%	<0.001
		Psychological health QOL	41.36	62.29	−33.60%	<0.001
		Social Relationships QOL	28.78	58.27	−50.61%	<0.001
		Environment QOL	29.25	62.61	−53.28%	<0.001
	>60 years	Physical health QOL	35.96	43.35	−17.05%	0.080
		Psychological health QOL	36.65	44.97	−18.50%	0.029
		Social Relationships QOL	23.16	39.08	−40.74%	<0.001
		Environment QOL	27.28	49.78	−45.20%	<0.001
TB relapses (*n* = 142)						
Age group	18–35 years	Physical health QOL	38.24	51.34	34.26%	<0.001
		Psychological health QOL	31.25	38.54	23.33%	<0.001
		Social Relationships QOL	37.58	43.75	16.42%	<0.001
		Environment QOL	29.19	35.16	20.45%	<0.001
	36–60 years	Physical health QOL	46.8	43.87	−6.26%	0.220
		Psychological health QOL	45.69	36.18	−20.81%	<0.001
		Social Relationships QOL	52.59	39.71	−24.49%	<0.001
		Environment QOL	55.71	36.58	−34.34%	<0.001
	>60 years	Physical health QOL	28.29	33.75	19.30%	0.032
		Psychological health QOL	32.05	36.25	13.10%	0.127
		Social Relationships QOL	22.35	26.67	19.33%	0.021
		Environment QOL	27.21	30.63	12.57%	0.103

In TB relapse cases, younger patients (18–35 years) with previous COVID-19 exhibited significantly lower QOL across all domains compared to those without prior infection (all *p* < 0.001). Among patients aged 36–60 years, significant reductions occurred in psychological (−20.81%), social relationships (−24.49%), and environmental (−34.34%) domains (all *p* < 0.001). Older relapse patients (>60 years) showed significant differences only in physical health (19.30%, *p* = 0.032) and social relationships (19.33%, *p* = 0.021).

Educational level influenced QOL outcomes distinctly among patients with and without previous COVID-19 (Supplementary Tables S1, S2), displaying complex interactions with gender and marital status. In patients without prior COVID-19, higher education generally improved QOL in married females, especially at secondary education levels, but led to notable declines for single females across psychological, social, and environmental domains. Single males mostly benefited from secondary education through better physical health and social relationships, whereas males living as married typically experienced reduced QOL.

Conversely, among patients with previous COVID-19, tertiary education notably worsened physical and environmental domains for single females but improved their social relationships, while single males significantly enhanced their physical and psychological health at this level. Married females with previous COVID-19 improved in physical and psychological health at secondary education, but experienced marked declines at tertiary levels, contrasting with married males who demonstrated improved social and environmental QOL but reduced psychological health at tertiary education. Divorced and widowed patients also showed contrasting trends, with widowed males gaining significantly across multiple domains post-COVID-19, whereas divorced males faced considerable social relationship declines. Thus, the presence or absence of previous COVID-19 substantially modified the relationship between educational attainment and QOL across demographic groups.

### 3.2 Logistic regression analysis

In the logistic regression model (Table 4), participants with a history of confirmed COVID-19 showed an estimated twofold increase in the odds of relapse (OR = 2.08, 95% CI = 1.29–3.37, *p* = 0.003). Age emerged as a strong predictor as well: individuals aged 36–60 years exhibited over a fivefold rise in relapse odds (OR = 5.08, 95% CI = 2.32–11.13, *p* < 0.001), and those older than 60 years experienced nearly a fourfold increase (OR = 3.96, 95% CI = 1.56–10.04, *p* = 0.004). Smoking was another significant risk factor, elevating relapse odds by about 76% (OR = 1.77, 95% CI = 1.15–2.70, *p* = 0.009). In contrast, completing secondary (OR = 0.48, 95% CI = 0.31–0.77, *p* = 0.002) or tertiary (OR = 0.46, 95% CI = 0.27–0.80, *p* = 0.006) education was associated with lower relapse likelihood. Neither gender nor marital status demonstrated a significant effect on relapse, and the constant term was very low (OR = 0.035, 95% CI = 0.0123–0.1004, *p* < 0.001), indicating minimal baseline risk when all variables were held at their reference levels.

**Table 4:** Marital status in without previous COVID-19

	Quality of life domains		Gender					
			Females (*n* = 224)			Females (*n* = 224)		
			Education level			Education level		
			Primary school (*n* = 10)	Primary school (*n* = 10)	Primary school (*n* = 10)	Primary school (*n* = 10)	Primary school (*n* = 10)	Primary school (*n*= 10)
Marital status	Single	Physical health	80.36	65.31 (–18.73%)	60.71 (–24.45%)	55.00	73.41 (33.47%)^*^	63.39 (15.25%)^*^
		Psychological health	85.42	53.87 (–36.94%)^**^	68.06 (–20.32%)^**^	55.83	67.13 (20.24%)	63.02 (12.88%)
		Social Relationships QOL	83.33	31.55 (–62.14%)^**^	69.44 (–16.67%)^*^	61.67	54.63 (–11.42%)	63.54 (3.03%)
		Environment QOL Score	84.38	63.39 (–24.88%)^*^	61.46 (–27.16%)^*^	52.5	67.01 (27.64%)^*^	62.89 (19.79%)^*^
	Married	Physical health	52.38	58.57 (11.82%)	57.14 (9.09%)	61.54	60.12 (–2.31%)	67.86 (10.27%)
		Psychological health	44.44	52.5 (18.14%)	58.33 (31.26%)	49.68	59.03 (18.82%)^**^	63.89 (28.60%)^**^
		Social Relationships QOL	55.56	53.33 (–4.01%)	41.67 (–25.00%)	62.18	62.22 (0.06%)	63.89 (2.75%)
		Environment QOL Score	53.13	60 (12.93%)	56.25 (5.87%)	54.33	63.23 (16.38%)^*^	68.75 (26.54%)^**^
	Living as married	Physical health	44.64	66.07 (48.01%)^*^	85.71 (92.00%)^*^	73.81	61.19 (–17.10%)	54.46 (–26.22%)
		Psychological health	43.75	56.25 (28.57%)	87.5 (100.00%)^*^	70.83	58.89 (–16.86%)	60.42 (–14.70%)
		Social Relationships QOL	50	62.5 (25.00%)	100 (100.00%)^*^	77.78	64.44 (–17.15%)	62.5 (–19.65%)
		Environment QOL Score	54.69	68.75 (25.71%)	84.38 (54.29%)	76.04	66.04 (–13.15%)	57.81 (–23.97%)
	Separated	Physical health	53.57	52.98 (–1.10%)	52.67 (–1.01%)	75	75 (0.00%)	42.86 (–42.85%)
		Psychological health	29.17	50 (71.41%)	70.45 (141.52%)^**^	70.83	71.18 (0.49%)	58.33 (–17.65%)
		Social Relationships QOL	25	51.39 (105.56%)^*^	50 (100.00%)^*^	50	58.33 (16.66%)	58.33 (16.66%)
		Environment QOL Score	40.63	52.08 (28.18%)	71.88 (76.91%)^*^	59.38	63.8 (7.44%)	75 (26.31%)
	Divorced	Physical health	64.29	57.14 (–11.12%)	62.35 (–3.00%)	51.34	55 (7.13%)	60.71 (18.25%)
		Psychological health	70.83	45.83 (–35.30%)^*^	60.42 (–14.70%)	66.67	58.33 (–12.51%)	45.83 (–31.26%)
		Social Relationships QOL	58.33	75 (28.58%)	66.67 (14.30%)	52.08	58.33 (12.00%)	50 (–3.99%)
		Environment QOL Score	65.63	68.75 (4.75%)	71.33 (8.68%)	58.2	54.38 (–6.56%)	65.63 (12.77%)
	Widowed	Physical health	42.86	64.29 (50.00%)^*^	17.86 (–58.33%)	51.43	69.05 (34.26%)	21.75 (–57.71%)^*^
		Psychological health	66.67	70.83 (6.24%)	20.83 (–68.76%)^*^	60.83	62.5 (2.75%)	23.11 (–62.01%)^*^
		Social Relationships QOL	41.67	58.33 (39.98%)	16.67 (–60.00%)	65	47.22 (–27.35%)	19.7 (–69.69%)^*^
		Environment QOL Score	56.25	65.63 (16.68%)	31.25 (–44.44%)	55	60.42 (9.85%)	34.38 (–37.49%)

*p < 0.05, **p < 0.01: statistical significant result.

**Figure 1:**
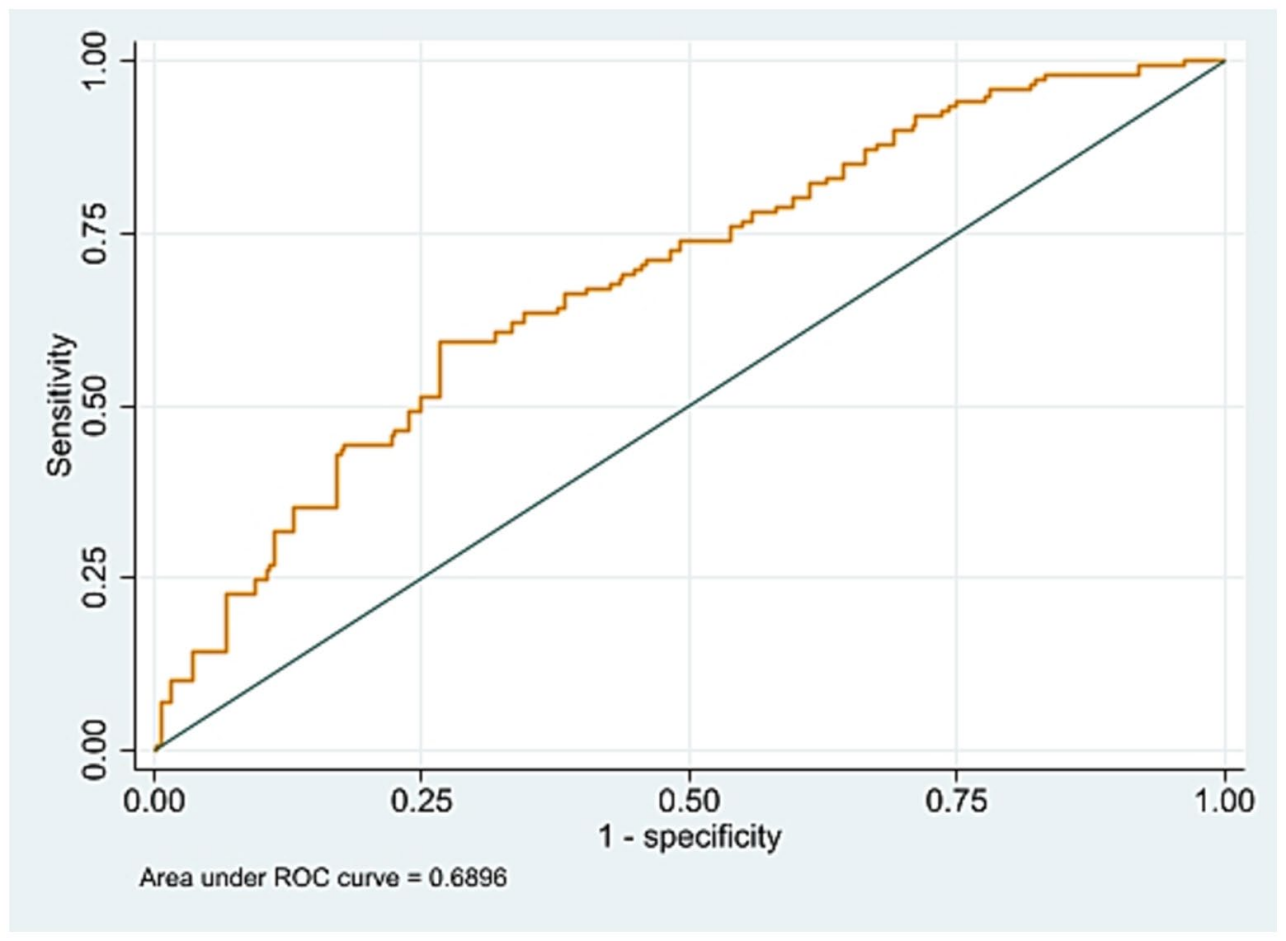
The receiver operating characteristic (ROC) curve of the binary logistic regression model for predicting tuberculosis relapse.

The receiver operating characteristic (ROC) curve (Figure 1) assessed the model performance and the discriminative ability of the logistic regression model predicting TB relapse. The area under the curve (AUC) is 0.6896, which suggests a moderate predictive performance, a value around 0.7 is considered acceptable.

### 3.3 Structural equation modeling

**Figure 2:**
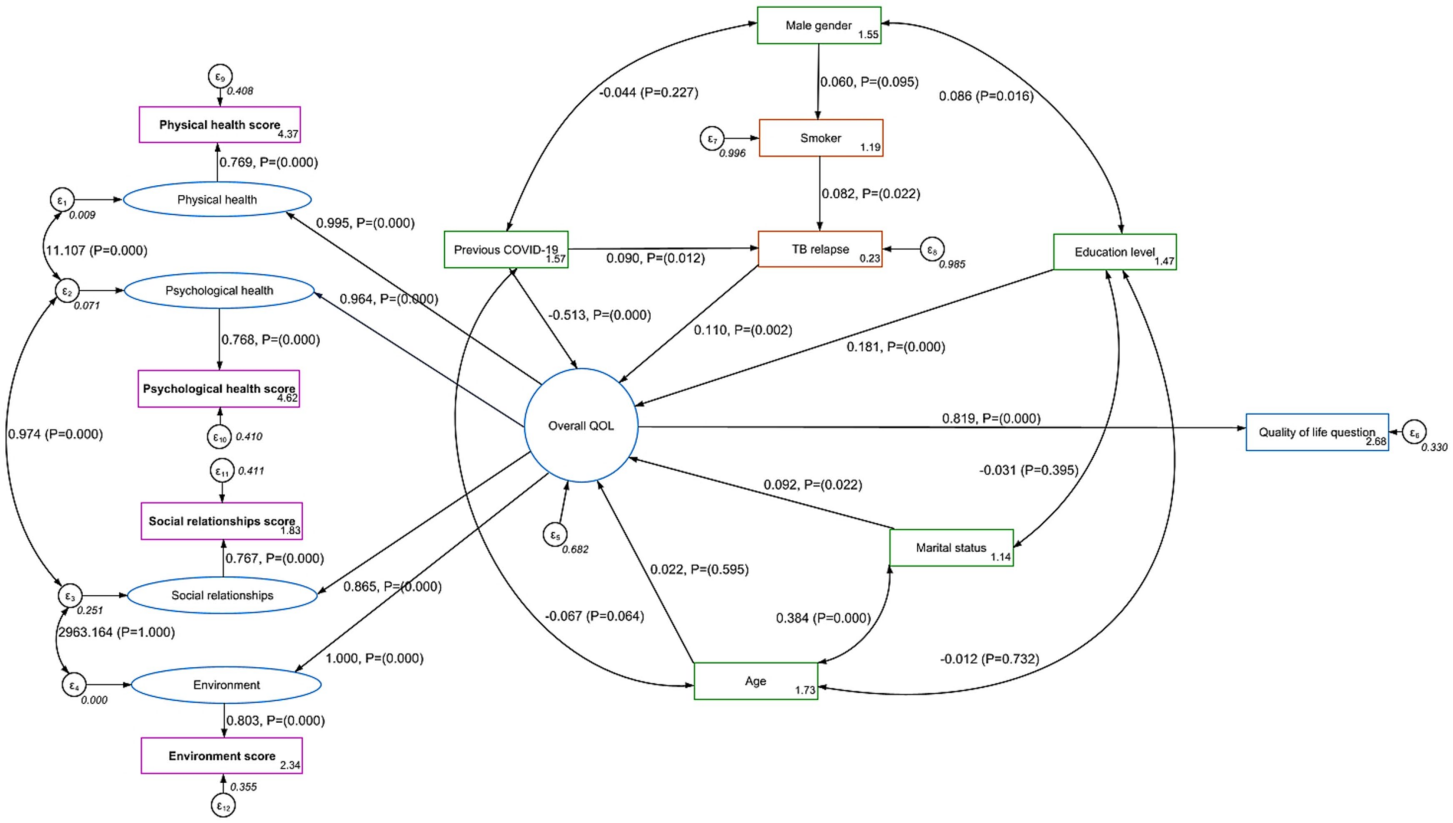
Structural equation model (SEM) illustrating how domain-specific latent constructs measured by the corresponding scores in the WHOQOL-BREF questionnaire (physical health, psychological health, social relationships, and environment) which contribute to Quality of Life (QOL) in tuberculosis patients. Predictors include marital status, COVID-19 status, TB relapse, gender, age, education, marital and smoking.

In structural equation modeling analysis (Figure 2), QOL was modeled as a higher-level latent factor measured by four domain-specific latent factors: physical health, psychological health, social relationships, and environment, as well as by a general QOL question (q1). Each domain factor was itself measured by one observed domain score (for example, the physical health latent factor was measured by the observed physical health score).

All four domain factors showed strong loadings onto the higher-order QOL factor: physical health (0.995), psychological health (0.964), social relationships (0.865) and environment (1.0) (the reference). Additionally, the general QOL item (q1) loaded at about 0.82 on the QOL factor.

These high loadings suggest that each of the four WHOQOL-like domains (physical, psychological, social, and environmental) plus the general QOL question strongly reflects an overarching construct of QOL.

On the structural paths predicting QOL, several variables emerged as significant. Marital status showed a small but positive association with QOL (*β* ≈ 0.09, *p* = 0.022), and education was also positively linked to QOL (*β* ≈ 0.18, *p* < 0.001). Relapse displayed a modest yet significant positive relationship (*β* ≈ 0.11, *p* = 0.002), whereas COVID status had a strong negative impact on QOL (β ≈ −0.51, *p* < 0.001). Age did not significantly affect QOL.

Regarding relapse, both smoking (β ≈ 0.08, p = 0.022) and COVID (β ≈ 0.09, *p* = 0.012) were significant risk factors. Meanwhile, smoking itself was marginally associated with gender (β ≈ 0.06, *p* = 0.095).

These results suggest that, in this sample of TB patients, COVID exerts one of the strongest negative influences on QOL, while marital status and education contribute positively. Relapse shows an unexpected positive association with QOL in this model (possibly reflecting complex health-service or coping factors), and smoking emerges as a notable factor linked to relapse.

## 4 Discussion

This study aimed to evaluate the impact of socio-demographic factors and COVID-19 history on the quality of life of TB patients, with a focused analysis on gender, marital status, and education level. TB significantly impacts the QOL of patients, affecting physical, mental, and social well-being. By examining the interplay between socio-demographic characteristics and prior COVID-19 infection, this research provides critical insights into how these factors influence TB patients’ health and highlights the need for targeted interventions to improve their overall QOL.

Our study did not directly evaluate QOL scores in the general population. However, available literature from Romania demonstrates that patients with TB consistently report significantly lower QOL compared to the general population. General Romanian population studies, utilizing validated QOL instruments such as the WHOQOL-BREF, EQ-5D-3L, and EQ-5D-5L, report pain/discomfort and mobility as major factors negatively influencing health utility, with certain health states rated worse than death by some respondents (27). Education, employment status, urban residency, and social support have been consistently identified as crucial determinants of higher QOL in the Romanian general population (28, 29). In particular, vulnerable groups such as pregnant women report improved physical and psychological well-being when possessing higher education levels, stable employment, partner support, and when delivering in private healthcare settings (29). The elderly with sarcopenia also report significantly compromised QOL, particularly affecting physical domains, as measured by the disease-specific SarQOL instrument (30, 31). External stressors, such as proximity to armed conflicts like the war in Ukraine, additionally exacerbate psychological distress and lower overall QOL among affected Romanians (32). Physical activity levels, frequently insufficient among Romanian youth, further influence perceived QOL (33). When comparing these data to TB patients, Margineanu et al. (22) documented notably lower scores across all QOL dimensions for both drug-susceptible and drug-resistant TB patients compared to national norms, highlighting severe impairment especially regarding pain, psychological distress, and social functioning. Similarly, another study identified significantly lower QOL scores among drug-resistant patients at baseline across physical, social, emotional, and occupational domains compared to their drug-sensitive counterparts, emphasizing the extensive impact of TB beyond clinical manifestations (21). These combined findings underscore the critical importance of incorporating comprehensive psychosocial and rehabilitative support into TB management programs to mitigate the substantial burden on patients’ lives.

QOL is a crucial indicator of overall health outcomes among patients with chronic diseases in Romania. Recent studies evaluating diverse patient groups including those with chronic kidney disease, diabetes, cancer, alcohol use disorder (AUD), and HIV highlight significant QOL impairments across multiple domains. For instance, patients undergoing hemodialysis frequently report lower physical and mental QOL compared to the general population, with work capability being notably impacted, particularly among older, female, or socioeconomically disadvantaged patients (34). Type 2 diabetic patients describe their condition as restrictive, notably in dietary freedom and leisure activities, and frequently report diminished QOL associated with older age, increased BMI, and barriers to adequate oral healthcare (35–38). Patients with laryngeal cancer, particularly those undergoing total laryngectomy, face considerable challenges in emotional, social, and functional domains, compounded by insomnia and financial stressors; despite the recognized benefits of psycho-oncologic interventions, their inconsistent implementation remains a challenge in Romania (39, 40). Individuals hospitalized for AUD consistently exhibit reduced QOL across physical, psychological, social, and environmental domains, with comorbidities such as hypertension further exacerbating these issues (41). Although people living with HIV generally report good QOL, high prevalence of anxiety and depression significantly impacts their overall well-being, especially among those with poor self-rated health, mental health issues, or unemployment (42). Recent validation of QOL measurement tools, such as SF-36, EQ-5D-3L/5L, and WHOQOL-BREF, along with development of Romanian-specific value sets, underscores efforts toward accurately assessing and addressing these QOL deficits, informing patient-centered care, and supporting effective health policy decisions (27).

TB prevalence is consistently higher in men than in women. Globally, case notifications reveal a male-to-female ratio of approximately 1.7 (43, 44). However, in low- and middle-income countries, the ratio is even more pronounced around 2.21 for bacteriologically positive TB cases (45). Our findings are consistent with this literature, revealing a persistent male predominance across all study groups.

Multiple studies have documented a significantly higher smoking prevalence among male TB patients with rates in some studies showing up to 61.7% of male TB patients being smokers compared to less than 10% among females a trend that aligns with our overall finding of male predominance (46–48). In the context of our study, which identified smoking as a significant predictor of TB relapse (OR = 1.77, *p* = 0.009), these data underscore the importance of nuanced, demographic-specific interventions. For instance, tailored smoking cessation programs could target married females who appear to have disproportionately higher smoking levels and single males, ensuring more effective mitigation of TB relapse risk. Furthermore, these results complement our broader observation that socio-demographic factors (including marital status and education) jointly shape both TB relapse risk and QOL outcomes in TB patients. Such insights can guide more integrated public health strategies that address smoking cessation, psychosocial support, and TB care concurrently.

This study examined demographic characteristics among TB patients based on previous COVID-19 infection and smoking status. Among new TB cases, smokers with prior COVID-19 infection were significantly younger than those without (43.36 ± 13.64 vs. 49.23 ± 14.19 years, *p* < 0.001). However, gender distribution did not differ significantly in relation to COVID-19 history for either smokers or non-smokers.

For TB relapse cases, neither age nor gender showed significant differences according to previous COVID-19 status among smokers or non-smokers. These findings suggest age-specific vulnerabilities, particularly among younger smokers newly diagnosed with TB, while demographic factors appear less influential in relapse cases. Such results underscore the need for targeted interventions addressing specific age groups and smoking behavior to improve patient outcomes.

The results of this study clearly demonstrate significant reductions in QOL scores among TB patients with prior COVID-19 infection compared to those without, across both newly diagnosed cases and relapse cases. Notably, among newly diagnosed TB patients, previous COVID-19 infection correlated with profound reductions in all measured QOL domains (physical health, psychological health, social relationships, and environmental quality), regardless of smoking status. This finding highlights the extensive impact COVID-19 can have, potentially exacerbating vulnerabilities already associated with TB.

Among smokers, the impairment was particularly pronounced in social relationships and environmental domains, suggesting that the combination of smoking and previous COVID-19 infection may create compounded psychosocial and environmental stressors. This aligns with existing literature that links smoking and chronic respiratory conditions to deteriorations in social interaction and environmental adaptability (8, 49, 50). In TB patients without previous COVID-19, smokers experienced lower psychological and social QOL, which supports studies showing that smoking cessation improves TB outcomes, including cure and relapse rates (11, 12). Similarly, an integrated TB-tobacco intervention in Malaysia led to notable QOL improvements (51).

Interestingly, among TB relapse patients, previous COVID-19 infection exhibited a differential impact dependent on smoking status. For smokers, the most significant effects were observed in psychological and social relationships, as well as environmental QOL, but not physical health. Non-smokers, however, showed consistently significant reductions across all QOL domains, underscoring the pervasive and lasting detrimental impact of COVID-19 on this subgroup. These nuanced differences may reflect distinct biological and behavioral responses, as smoking could potentially mask or alter symptom perception or reporting in physical health domains.

The consistent and significant reduction in environmental and social relationship QOL domains across both groups underscores the broader societal and psychosocial disruptions associated with COVID-19, possibly exacerbated in populations already vulnerable due to TB (13, 52–54). This finding suggests the necessity of integrated psychosocial support and environmental interventions within TB management programs, particularly tailored to individuals with a history of COVID-19 infection.

Our findings indicate a marked reduction in QOL among TB patients who previously had COVID-19, with important variations by age. For newly diagnosed TB patients, the adverse impact of prior COVID-19 infection was significant across all QOL domains, especially pronounced in younger (18–35 years) and middle-aged (36–60 years) individuals. The largest deficits were observed in environmental and social relationship domains, suggesting that younger and middle-aged patients experience greater disruption in social functioning and perceived quality of their living conditions following COVID-19 infection.

Older patients (>60 years) with new TB diagnoses showed significant impairment predominantly in the social and environmental domains, with less pronounced differences in physical and psychological domains. This could imply resilience or adaptive mechanisms regarding physical and psychological health in older patients, while social isolation and environmental restrictions post-COVID-19 remain impactful across all age groups.

Among patients experiencing TB relapse, younger individuals demonstrated universally lower QOL scores in all domains after prior COVID-19 infection, highlighting heightened vulnerability and significant psychosocial disruption within this subgroup. Middle-aged relapse patients experienced substantial impairments in psychological, social, and environmental aspects, suggesting persistent psychosocial stressors associated with COVID-19 sequelae, despite relatively stable physical health scores. Older relapse patients exhibited fewer significant QOL differences, primarily in physical health and social relationships, indicating a potentially smaller impact or better coping strategies in psychological and environmental domains among elderly patients.

Marital status and education markedly affect QOL among TB patients. For single individuals, females with secondary and tertiary education experienced significant declines in psychological (−36.94%) and social (−62.14%) QOL, while single males with secondary education showed improved physical health (+33.47%). Among married patients, females with secondary education had significant gains in physical, psychological, and environmental domains, although tertiary education was linked to a notable decline in social relationships, whereas married males benefited from tertiary education with significant physical and psychological improvements. This contrasts with literature suggesting that higher education generally enhances social relationships and emotional well-being through improved communication skills and social networks (55–57). Overall, our results largely align with previous studies showing that marriage and education provide emotional and social support to improve QOL (57–59), yet they also suggest that the benefits of education may vary by gender and marital status, emphasizing the need for tailored interventions.

TB patients with confirmed previous COVID-19 reveal complex interactions between marital status, education, and gender on QOL. Single females with tertiary education experienced significant declines in physical and environmental QOL, while single males with tertiary education showed improvements in physical and psychological domains. In married patients, females with secondary education had significant gains in physical and psychological health, whereas married males benefited from tertiary education. These nuanced outcomes contrast with a Greek study reporting higher QOL in females, married, highly educated, and non-smokers (60) and with data from Guinea showing overall lower QOL due to financial and nutritional stressors (61). Furthermore, the 13% mortality rate observed in TB/COVID-19 co-infection in India and China (62) underscores the importance of addressing socio-demographic factors in treatment strategies. Overall, our results highlight the need for tailored interventions that consider marital status and education to improve QOL in TB patients, particularly those with a history of COVID-19.

Our logistic regression findings emphasize that a confirmed history of COVID-19 doubles the odds of TB relapse (OR = 2.08, *p* = 0.003), which aligns with prior reports indicating that TB incidence increases in COVID-19 survivors due to the immunosuppressive effects of the virus and its treatment (16).

The vulnerability of TB patients is further heightened by the presence of coinfections, particularly viral infections like SARS-CoV-2, which complicate clinical presentation, delay diagnosis, and worsen treatment outcomes. This is particularly relevant in low- and middle-income countries (LMICs), where healthcare access barriers, socioeconomic challenges, and a high burden of infectious diseases collectively amplify the negative impact on TB management (63). Evidence emphasizes the urgent need for integrated diagnostic strategies and optimized treatment approaches to address coinfections and improve overall health outcomes in TB patients. Our findings further underscore a clinically relevant interaction between previous COVID-19 infection and increased TB relapse risk. Prior SARS-CoV-2 infection likely exacerbates the vulnerability of TB patients to relapse through mechanisms such as residual pulmonary damage, persistent immunosuppressive effects, and potential delays in TB diagnosis or treatment initiation during the pandemic period. These observations highlight the necessity for enhanced post-COVID-19 monitoring and tailored interventions in TB care pathways. This clinically intuitive link aligns with recent literature indicating that patients recovering from COVID-19 may exhibit prolonged impairment of pulmonary and immune function, significantly increasing the risk for reactivation or recurrence of TB (54, 64). These insights highlight the need for enhanced surveillance and targeted clinical interventions in TB patients with a history of COVID-19, aiming to minimize relapse rates and improve patient outcomes.

Age also emerged as a strong predictor in our model, with individuals aged 36–60 having over a fivefold increase (OR = 5.08, *p* < 0.001) and those >60 nearly fourfold (OR = 3.96, *p* = 0.004), compared with 18–35 years age group. The higher relapse risk in older ages is consistent with studies showing that older age is a key factor in TB relapse, as observed in hospital-based case–control studies (65). Additionally, our observation that smoking elevates relapse odds by 76% (OR = 1.77, *p* = 0.009) is in agreement with epidemiological analyses from cohort studies which have reported that heavy smoking significantly increases TB recurrence risk, sometimes by more than twofold (66, 67). Our findings indicate that higher education serves as a protective factor against TB relapse, with secondary and tertiary education significantly reducing relapse odds (OR = 0.48, *p* = 0.002 and OR = 0.46, *p* = 0.006, respectively). This aligns with previous studies reporting that TB patients with lower education levels exhibit a higher relapse rate (12.2%) compared to those with higher education (7.1%), which can be related to lower adherence to TB treatment in people with lower education (that includes lower health education) (68).

Consistent with our findings, previous population-based studies reinforce the significant association between smoking and increased risk of TB recurrence after successful treatment. For instance, a study found that individuals who smoked more than 10 cigarettes per day had double the recurrence risk compared to non-smokers (adjusted HR = 2.0).

Research consistently shows a strong association between smoking and reduced QOL across all age groups, although the extent of its impact varies with age. In young and middle-aged adults, smoking especially heavy smoking is clearly linked to significantly lower QOL scores, with increased numbers of physically and mentally unhealthy days compared to non-smokers (69, 70). In older adults, the relationship becomes more complex; while some studies report slightly higher QOL scores among moderate older smokers, potentially due to survivor bias, heavy smoking remains strongly associated with worse outcomes, particularly affecting social participation and psychological well-being (69, 71, 72).

This increased risk highlights the necessity of integrating smoking cessation interventions into TB treatment programs as a strategy to prevent relapse (67). Furthermore, a TB control study involving 16,345 patients reported a clear gradient in relapse risk from never-smokers to ex-smokers and current smokers, with hazard ratios of 1.00, 1.33, and 1.63, respectively. Approximately 19.4% of TB relapses in this population were directly attributable to smoking. These findings strongly support the inclusion of targeted smoking cessation measures in comprehensive TB control strategies (8).

Gender and marital status did not significantly affect relapse risk in our regression analysis, which may indicate that when controlling for clinical and socioeconomic variables, these factors exert less influence, compared with univariate analysis. Overall, our results corroborate and expand on previous findings, emphasizing the compounded risks of COVID-19, older age, and smoking on TB relapse while highlighting the protective role of education (16, 65–67, 73).

Complementing these findings, our structural equation model demonstrated that the four WHOQOL-BREF domains, physical, psychological, social, and environmental along with a general QOL question, strongly contribute to an overarching QOL construct, with high factor loadings (0.865–1.0). On the structural paths, marital status (*β* ≈ 0.09, *p* = 0.022) and education (β ≈ 0.18, *p* < 0.001) were positively associated with QOL, while COVID status had a strong negative effect (β ≈ −0.51, *p* < 0.001).

Our finding that married and higher-educated individuals report better QoL aligns with broader patterns observed in general populations, as marriage and education typically enhance psychosocial support networks, socioeconomic stability, and access to healthcare resources. Previous studies have repeatedly shown that being married and having higher educational attainment are associated with improved physical and psychological well-being, especially among men, likely due to increased social support and health literacy (55, 57). However, within the specific context of TB these socio-demographic factors may have an even more pronounced impact on patient outcomes due to their potential influence on treatment adherence, health behaviors, and coping strategies when facing a chronic, socially stigmatizing disease.

In terms of relapse risk, both smoking (*β* ≈ 0.08, *p* = 0.022) and COVID (β ≈ 0.09, *p* = 0.012) emerged as significant contributors, and smoking was marginally linked to gender. Overall, these results suggest that, among TB patients, COVID-19 is a major detractor from QOL, while supportive factors such as marital status and education can mitigate some negative impacts.

Our findings underscore the multifaceted impact of TB on patients’ QOL, revealing significant impairment across physical, psychological, social, and occupational domains. The exacerbation of these impairments by factors such as prior COVID-19 infection, advanced age, smoking, lower education, and socioeconomic vulnerability emphasizes the need for comprehensive, multidisciplinary approaches in TB care. Enhancing patient outcomes requires targeted strategies that integrate medical interventions with psychosocial support, smoking cessation programs, and educational initiatives tailored to patients’ specific demographic and clinical profiles. Management strategies for TB patients should incorporate systematic screening and management of comorbid conditions, such as diabetes mellitus, along with the use of early predictive biomarkers to identify high-risk individuals, both of which have been shown to significantly influence treatment outcomes and overall quality of life (38, 74). Future research should expand on these findings through longitudinal studies to better understand evolving patient needs and inform effective, context-specific public health policies. Ultimately, a holistic approach addressing both clinical treatment and quality-of-life improvements will be pivotal in advancing TB care, reducing relapse risks, and alleviating the broader societal burden of this disease.

### 4.1 Future directions

Future research should employ longitudinal study designs to better elucidate causal relationships between COVID-19 history, socio-demographic factors, and QOL among TB patients. Expanding the sample size and including multi-center studies across different regions and healthcare settings will enhance the generalizability of the findings. Additionally, integrating more detailed assessments of socio-economic status such as income levels, nutritional status, and access to healthcare services could further clarify the complex interactions influencing patient outcomes. Investigating the long-term effects of COVID-19 on TB patients’ QOL, including potential changes over time and the impact of ongoing public health interventions, is also warranted. Finally, exploring targeted interventions, such as smoking cessation programs and tailored socio-economic support, may provide practical insights into improving both clinical outcomes and QOL in this high-risk population.

### 4.2 Limitations

This study has several limitations. The reliance on self-reported socio-demographic and lifestyle data, such as smoking, may introduce recall and reporting biases. The study was conducted in a specific geographic region (South-Western Romania), which may limit the generalizability of the results to other regions or populations with different socio-economic and healthcare contexts. The subgroup analyses comparing patients with and without previous COVID-19 infection occasionally involved small sample sizes, limiting the statistical power and generalizability of the findings. Although we adjusted for several confounders, including age, education, and smoking status, residual confounding by unmeasured factors such as nutritional status or TB treatment adherence cannot be ruled out. Moreover, the severity of prior COVID-19 infections was not consistently documented, which could affect the associations observed with TB relapse and QOL.

QOL assessment was cross-sectional, and the duration between recovery from COVID-19 and QOL evaluation varied among participants; thus, long-term follow-up data were not available. While all participants analyzed for relapse had documented completion of anti-TB treatment, precise adherence levels and potential residual effects of treatment were not individually assessed, potentially influencing relapse and QOL outcomes. Although rigorous exclusion criteria were applied, undetected past COVID-19 infections without laboratory confirmation remain a possibility, potentially impacting group classifications and study results. Despite these limitations, our findings provide important insights into the interplay between COVID-19, socio-demographic factors, and TB outcomes, highlighting the need for integrated interventions targeting these high-risk groups.

## 5 Conclusion

TB patients with a history of COVID-19, especially smokers, experience a marked decline in QOL. COVID-19, advanced age, and smoking significantly increase the risk of TB relapse, while higher education appears to have a protective effect. These results underscore the need to incorporate COVID-19 prevention, focused smoking cessation efforts, and socio-economic interventions into TB management programs not only to reduce relapse rates but also to enhance overall QOL in high-risk patient groups. Further research is essential to clarify the underlying mechanisms and to develop tailored strategies that address both clinical outcomes and QOL improvements.

## Data Availability

The raw data supporting the conclusions of this article will be made available by the authors, without undue reservation.
