# Supplementary material for: From pandemic to endemic: assessing the impact of COVID-19 history and socio-demographic factors on quality of life in tuberculosis patients
Source: Front Med (Lausanne). 2025 Jun 25;12:1582726. doi: 10.3389/fmed.2025.1582726 (PMC12239877; doi:10.3389/fmed.2025.1582726)
Supplement: Supplementary file 1 [file Table_1.DOCX]

Supplementary Table S1. Marital status in without previous COVID-19

| **Marital status** |  | | Gender | | | | | |
| --- | --- | --- | --- | --- | --- | --- | --- | --- |
|  |  |  | Females  (n=224) | | | Males  (n=539) | | |
|  |  |  | Education level | | | Education level | | |
|  |  |  | Primary school  (n=10) | Secondary  school  (n=29) | Tertiary school  (n=19) | Primary school  (n=44) | Secondary school  (n=83) | Tertiary school  (n=36) |
|  | Single | Physical health | 80.36 | 65.31(-18.73%) | 60.71(-24.45%) | 55.00 | 73.41(33.47%)^*^ | 63.39(15.25%)^*^ |
|  |  | Psychological health | 85.42 | 53.87(-36.94%)^**^ | 68.06(-20.32%)^**^ | 55.83 | 67.13(20.24%) | 63.02(12.88%) |
|  |  | Social Relationships QOL | 83.33 | 31.55(-62.14%)^**^ | 69.44(-16.67%)^*^ | 61.67 | 54.63(-11.42%) | 63.54(3.03%) |
|  |  | Environment QOL Score | 84.38 | 63.39(-24.88%)^*^ | 61.46(-27.16%)^*^ | 52.5 | 67.01(27.64%)^*^ | 62.89(19.79%)^*^ |
|  |  |  |  |  |  |  |  |  |
|  | Married | Physical health | 52.38 | 58.57(11.82%) | 57.14(9.09%) | 61.54 | 60.12(-2.31%) | 67.86(10.27%) |
|  |  | Psychological health | 44.44 | 52.5(18.14%) | 58.33(31.26%) | 49.68 | 59.03(18.82%)^**^ | 63.89(28.60%)^**^ |
|  |  | Social Relationships QOL | 55.56 | 53.33(-4.01%) | 41.67(-25.00%) | 62.18 | 62.22(0.06%) | 63.89(2.75%) |
|  |  | Environment QOL Score | 53.13 | 60(12.93%) | 56.25(5.87%) | 54.33 | 63.23(16.38%)^*^ | 68.75(26.54%)^**^ |
|  |  | |  |  |  |  |  |  |
|  | Living as married | Physical health | 44.64 | 66.07(48.01%)^*^ | 85.71(92.00%)^*^ | 73.81 | 61.19(-17.10%) | 54.46(-26.22%) |
|  |  | Psychological health | 43.75 | 56.25(28.57%) | 87.5(100.00%)^*^ | 70.83 | 58.89(-16.86%) | 60.42(-14.70%) |
|  |  | Social Relationships QOL | 50 | 62.5(25.00%) | 100(100.00%)^*^ | 77.78 | 64.44(-17.15%) | 62.5(-19.65%) |
|  |  | Environment QOL Score | 54.69 | 68.75(25.71%) | 84.38(54.29%) | 76.04 | 66.04(-13.15%) | 57.81(-23.97%) |
|  |  | |  |  |  |  |  |  |
|  | Separated | Physical health | 53.57 | 52.98(-1.10%) | 52.67(-1.01%) | 75 | 75(0.00%) | 42.86(-42.85%) |
|  |  | Psychological health | 29.17 | 50(71.41%) | 70.45(141.52%)^**^ | 70.83 | 71.18(0.49%) | 58.33(-17.65%) |
|  |  | Social Relationships QOL | 25 | 51.39(105.56%)^*^ | 50(100.00%)^*^ | 50 | 58.33(16.66%) | 58.33(16.66%) |
|  |  | Environment QOL Score | 40.63 | 52.08(28.18%) | 71.88(76.91%)^*^ | 59.38 | 63.8(7.44%) | 75(26.31%) |
|  |  | |  |  |  |  |  |  |
|  | Divorced | Physical health | 64.29 | 57.14(-11.12%) | 62.35(-3.00%) | 51.34 | 55(7.13%) | 60.71(18.25%) |
|  |  | Psychological health | 70.83 | 45.83(-35.30%)^*^ | 60.42(-14.70%) | 66.67 | 58.33(-12.51%) | 45.83(-31.26%) |
|  |  | Social Relationships QOL | 58.33 | 75(28.58%) | 66.67(14.30%) | 52.08 | 58.33(12.00%) | 50(-3.99%) |
|  |  | Environment QOL Score | 65.63 | 68.75(4.75%) | 71.33(8.68%) | 58.2 | 54.38(-6.56%) | 65.63(12.77%) |
|  |  | |  |  |  |  |  |  |
|  | Widowed | Physical health | 42.86 | 64.29(50.00%)^*^ | 17.86(-58.33%) | 51.43 | 69.05(34.26%) | 21.75(-57.71%)^*^ |
|  |  | Psychological health | 66.67 | 70.83(6.24%) | 20.83(-68.76%)^*^ | 60.83 | 62.5(2.75%) | 23.11(-62.01%)^*^ |
|  |  | Social Relationships QOL | 41.67 | 58.33(39.98%) | 16.67(-60.00%) | 65 | 47.22(-27.35%) | 19.7(-69.69%)^*^ |
|  |  | Environment QOL Score | 56.25 | 65.63(16.68%) | 31.25(-44.44%) | 55 | 60.42(9.85%) | 34.38(-37.49%) |

^*^ - p<0.05, ^**^ - p<0.01, ^***^ - p<0.001

Supplementary Table S2. Marital status in confirmed previous COVID-19

| **Marital status** |  | | Gender | | | | | |
| --- | --- | --- | --- | --- | --- | --- | --- | --- |
|  |  |  | Females  (n=224) | | | Males  (n=539) | | |
|  |  |  | Education level | | | Education level | | |
|  |  |  | Primary school  (n=54) | Secondary  school  (n=92) | Tertiary school  (n=20) | Primary school  (n=70) | Secondary school  (n=220) | Tertiary school  (n=86) |
|  | Single | Physical health | 35.71 | 34.02(-4.73%) | 27.23(-23.75%) | 40.71 | 39.34(-3.37%) | 48(17.91%) |
|  |  | Psychological health | 38.54 | 38.82(0.73%) | 34.37(-10.82%) | 37.08 | 40.89(10.28%) | 46(24.06%) |
|  |  | Social Relationships QOL | 28.33 | 28.95(2.19%) | 40.62(43.38%)^*^ | 32.5 | 29.3(-9.85%) | 33.67(3.60%)^*^ |
|  |  | Environment QOL Score | 31.56 | 26.48(-16.10%) | 21.09(-33.17%) | 32.81 | 27.2(-17.10%)^*^ | 38.75(18.10%)^*^ |
|  |  |  |  |  |  |  |  |  |
|  | Married | Physical health | 35.71 | 43.13(20.78%) | 29.76(-16.66%) | 39.52 | 40.87(3.42%) | 34.4(-12.96%) |
|  |  | Psychological health | 33.33 | 43.91(31.74%) | 23.61(-29.16%)^*^ | 35.28 | 42.02(19.10%)^*^ | 31.36(-11.11%)^**^ |
|  |  | Social Relationships QOL | 39.58 | 29.17(-26.30%) | 22.22(-43.86%) | 21.11 | 36.9(74.80%)^***^ | 26.32(24.68%)^***^ |
|  |  | Environment QOL Score | 28.91 | 31.01(7.26%) | 40.63(40.54%) | 27.29 | 34.42(26.13%)^*^ | 30.43(11.51%)^*^ |
|  |  | |  |  |  |  |  |  |
|  | Living as married | Physical health | 39.29 | 42.11(7.18%) | 39.29(0.00%) | 36.34 | 37.01(1.84%) | 28.93(-20.39%) |
|  |  | Psychological health | 50 | 44.74(-10.52%) | 43.75(-12.50%) | 32.11 | 33.96(5.76%) | 40(24.57%) |
|  |  | Social Relationships QOL | 50 | 32.89(-34.22%)^**^ | 29.17(-41.66%)^**^ | 18.14 | 22.73(25.30%) | 27.5(51.60%) |
|  |  | Environment QOL Score | 50 | 35.69(-28.62%)^**^ | 34.38(-31.24%)^*^ | 22.61 | 27.18(20.21%) | 29.69(31.31%) |
|  |  | |  |  |  |  |  |  |
|  | Separated | Physical health | 46.43 | 46.77(0.70%) | 45.03(-3.00%) | 35.71 | 44.22(23.83%) | 44.64(25.01%) |
|  |  | Psychological health | 40.28 | 45.83(13.78%) | 37.5(-6.90%) | 36.11 | 37.1(2.74%) | 35.42(-1.91%) |
|  |  | Social Relationships QOL | 38.89 | 33.33(-14.30%) | 33.33(-14.30%) | 30.56 | 29.37(-3.89%) | 29.17(-4.55%) |
|  |  | Environment QOL Score | 33.33 | 31.25(-6.24%) | 31.25(-6.24%) | 28.13 | 28.87(2.63%) | 34.38(22.22%) |
|  |  | |  |  |  |  |  |  |
|  | Divorced | Physical health | 37.66 | 50.68(34.57%)^**^ | 51.79(37.52%)^**^ | 43.18 | 40.41(-6.42%) | 43.71(1.23%) |
|  |  | Psychological health | 37.12 | 35.32(-4.85%) | 37.5(1.02%) | 41.67 | 43.2(3.67%) | 41.67(0.00%) |
|  |  | Social Relationships QOL | 27.27 | 34.92(28.05%) | 41.67(52.81%)^*^ | 40.91 | 20.61(-49.62%)^*^ | 28.33(-30.75%)^**^ |
|  |  | Environment QOL Score | 34.38 | 31.7(-7.80%) | 39.06(13.61%) | 31.53 | 27.47(-12.88%) | 22.88(-27.43%) |
|  |  | |  |  |  |  |  |  |
|  | Widowed | Physical health | 34.52 | 32.14(-6.89%) | 35.71(3.45%) | 36.73 | 54.67(48.84%)^*^ | 42.14(14.73%)^*^ |
|  |  | Psychological health | 31.94 | 39.58(23.92%) | 33.33(4.35%) | 38.99 | 52.56(34.80%)^*^ | 49.17(26.11%) |
|  |  | Social Relationships QOL | 31.48 | 30.56(-2.92%) | 16.67(-47.05%)^*^ | 17.26 | 34.62(100.58%)^*^ | 35(102.78%)^**^ |
|  |  | Environment QOL Score | 24.31 | 33.85(39.24%)^*^ | 25(2.84%) | 25.67 | 36.3(41.41%)^**^ | 41.25(60.69%)^*^ |

^*^ - p<0.05, ^**^ - p<0.01, ^***^ - p<0.001
